# The Nuss procedure for pectus excavatum: An effective and safe approach using bilateral thoracoscopy and a selective approach to use multiple bars in 296 adolescent and adult patients

**DOI:** 10.1371/journal.pone.0233547

**Published:** 2020-05-29

**Authors:** Po-Cheng Lo, I-Shiang Tzeng, Min-Shiau Hsieh, Mei-Chen Yang, Bo-Chun Wei, Yeung-Leung Cheng

**Affiliations:** 1 Division of Thoracic Surgery, Department of Surgery, Taipei Tzu Chi Hospital, Buddhist Tzu Chi Medical Foundation, New Taipei City, Taiwan; 2 Department of Research, Taipei Tzu Chi Hospital, Buddhist Tzu Chi Medical Foundation, New Taipei City, Taiwan; 3 Division of Pulmonary Medicine, Department of Internal Medicine, Taipei Tzu Chi Hospital, Buddhist Tzu Chi Medical Foundation, New Taipei City, Taiwan; 4 School of Medicine, Tzu Chi University, Hualien, Taiwan; Cleveland Clinic, UNITED STATES

## Abstract

The Nuss procedure is a minimally invasive repair used to treat pectus excavatum. A bilateral thoracoscopy-assisted approach has been reported as a safe method for Nuss repair. The aim of this observational cohort study is to evaluate the application of the bilateral thoracoscopy-inspection to assist in the selection of the number of bars for correction of the pectus deformity in adolescents and adults. A retrospective chart review was performed on all adolescent and adult patients (296 patients: 257 male, 39 female; aged of 23.9 ± 7.7 years) with pectus excavatum primarily corrected with the modified Nuss repair from August 2014 to January 2018. The patients were divided into three age groups (A: 12 years ≦ age < 19 years, n = 73; B: 19 years ≦ age < 30 years, n = 175; C: age ≧ 30 years, n = 48). Advanced repair of deformed chest walls using more than one bar depended on the change detected via gross and perioperative thoracoscopy-inspection. The results showed that two or three pectus bars were used in 268 patients (90.5%). The overall complication rate after a postoperative follow-up of 28.6 ± 11 months was 6.8% (20/296), without mortality, major bleeding, or serious infectious complications. A multivariate logistic regression analysis showed that the complications were only associated with Haller index (adjusted OR = 1.2935, p = 0.0317) under controlling confounding factors. The postoperative sternovertebral distance was significantly improved from 7.3±1.6 to 10.1± 2.8 cm (p<0.001). The thoracoscopy-assisted approach of Nuss repair for correction of pectus excavatum was safe and effective approach and could also be used as an alternative approach for the selection of placed bars in adolescent and adult patients. Further studies regarding long-term outcomes are required.

## Introduction

Pectus excavatum (PE), a common congenital chest wall deformity, usually presents in the neonatal period but can also occur during puberty [1,2]. As the child enters puberty, symptoms of cardiopulmonary compression may develop. Surgical correction is still considered the standard treatment for PE. In 1998, Nuss et al [3] documented a minimally invasive method for the correction of PE with good results. The procedure initially involves the introduction of a curved, stainless-steel bar behind the sternum to correct the depressed chest wall without resection of the costal cartilages. The technique has been widely developed and has been used extensively in children, adolescents, and adults over the past decade [4–9].

Despite universal acceptance, the procedure has several postoperative complications (about 10%-50%) including rare life-threatening complications [5–8]. Risk of complications increases with patient age or with the use of more than one pectus bar [4,7–9]. Several modifications to minimize the risk of the Nuss procedure have been introduced. The application of the thoracoscope in Nuss repair demonstrated an increased safety of the operation [2,5,9,10,11]. We previously demonstrated that the modified bilateral thoracoscopy-assisted approach in Nuss repair could permit direct inspection in the mediastinum and also facilitate mediastinal dissection while eliminating the risk of cardiopulmonary injuries [9,11].

Due to the popularity of the Nuss procedure and the accumulation of surgical experience, the increased use of more than one bar for correction of the sunken chest wall has also been reported recently, especially in adult patients [4–6,8,9,12]. The number of bars used in Nuss surgery is primarily determined by the gross appearance of the deformity. But in some cases, such as in women (because of the breasts) and obese patients, the deformity of the bone cage is not completely consistent with the external deformity of the chest wall [13,14].

In recent years we further observed the intraoperative change of the inner chest wall under thoracoscopic inspection during insertion of the bar(s) to help determine the number of bar(s) used for Nuss repair of PE. An observational cohort study of patients with pectus excavatum after surgical correction was performed. Demographics, clinical characteristics, surgical data and results were analyzed and discussed below.

## Materials and methods

### Patients

This study was approved by the Ethics Committee and the Institutional Review Board of the Taipei Tzu-Chi Hospital, Taipei, Taiwan, ROC (IRB No: 07-X-036). Patient consent was waived by the IRB because of the retrospective nature of the study. We included all adolescent and adult patients (aged 12 years and older) with PE who underwent surgical repair at our hospital from August 2014 to January 2018, with follow-up until July 2018; patients should be followed at least 6 months after operation [3, 8, 12]. Evaluations were conducted by taking complete history and physical examination, chest radiography, electrocardiography, pulmonary function tests (PFT), echocardiography, and computed tomography (CT) scanning of the chest were all performed. The Haller index (HI) on the preoperative CT scan [15] and sternovertebral distance (SVD) on the pre- and postoperative lateral chest radiographs [10] were determined. The overall complications, and postoperative follow-up data were collected and assessed.

**Fig 1:**
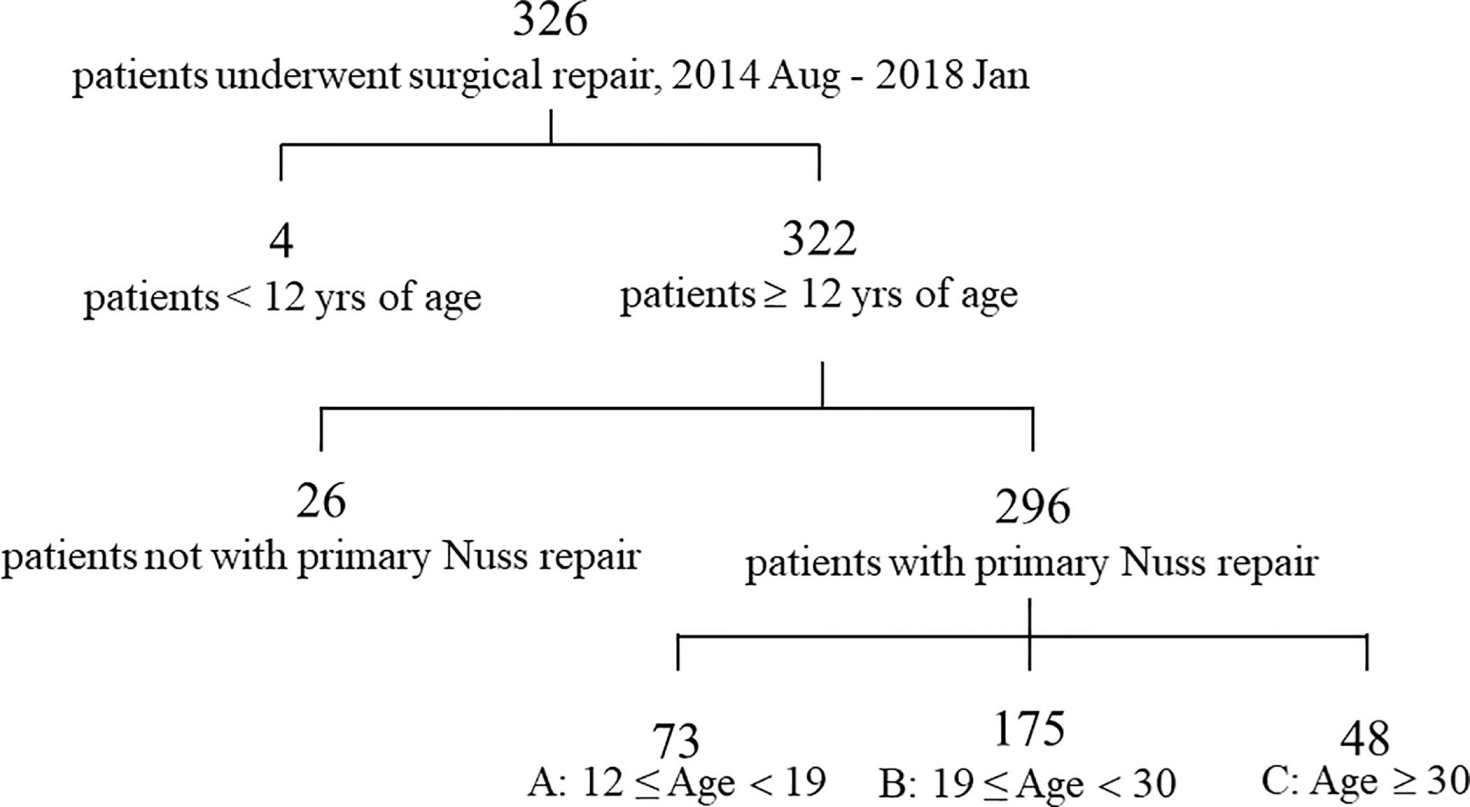
Flow chart of patient selection.

A retrospective chart review of all patients at our institute who underwent the Nuss procedure from 2014 to 2018 yielded 326 patients, of whom 296 were included in this study based on age and exclusion of children and patients where similar procedures were performed before (Fig 1). The primary indications for surgical repair were presence of two or more of the following criteria: (1) progression of the deformity; (2) exercise intolerance; (3) progressive chest pain or dyspnea; (4) restrictive ventilatory impairment; (5) HI >3.25; (6) cardiac compression; and (7) mitral valve prolapse [9,16]. Patients were divided into the following three age groups: A (adolescence): 12 years ≦ age < 19 years, n = 73; B (young adults): 19 years ≦ age < 30 years, n = 175; C (old adults): age ≧30 years, n = 48).

### Surgical preparation and procedure (see S1 Video)

A modified bilateral thoracoscopy-assisted Nuss repair procedure (with right-to-left mediastinal dissection) was performed as previously described [9]. The chief modification of this technique was that the bar(s) used depended on the intraoperative chest wall changes monitored by thoracoscopic inspection and on gross appearance.

**Fig 2:**
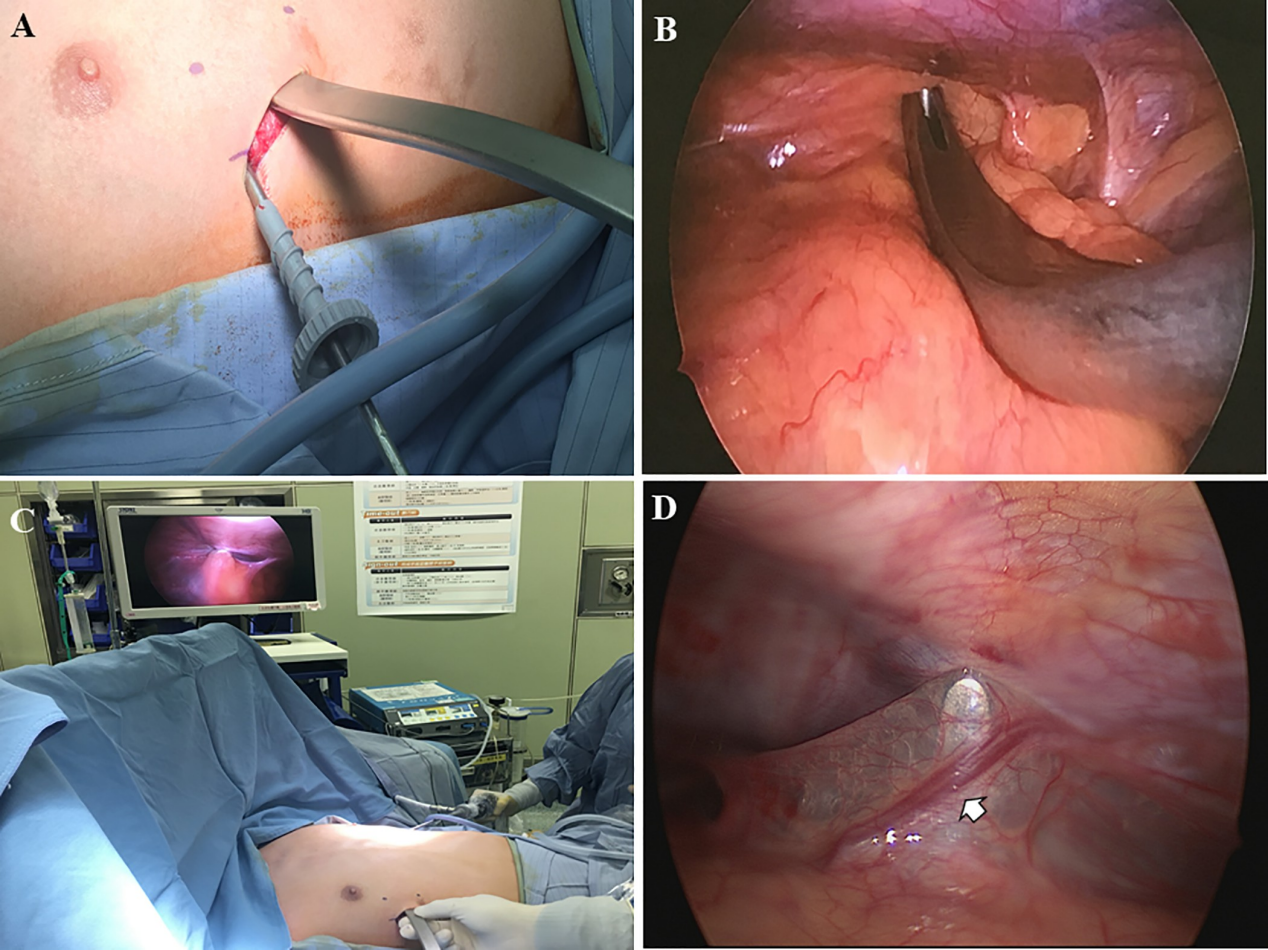
Bilateral uniport thoracoscope-assisted technique for mediastinal dissection. (A) A 30° 5-mm thoracoscope was inserted via a 3-cm skin incision on the right lateral chest wall and the introducer was applied for mediastinal dissection (B) Checking of the pleural cavity and mediastinum and a right-to-left mediastinal dissection under direct thoracoscopic inspection (C) After the tip of the introducer crossed the midline, the thoracoscope was shifted to the wound on the left side of the chest and a substernal tunnel was created (D) Aberrant vessels in the mediastinal pleura were avoided to prevent injury during mediastinal dissection.

**Fig 3:**
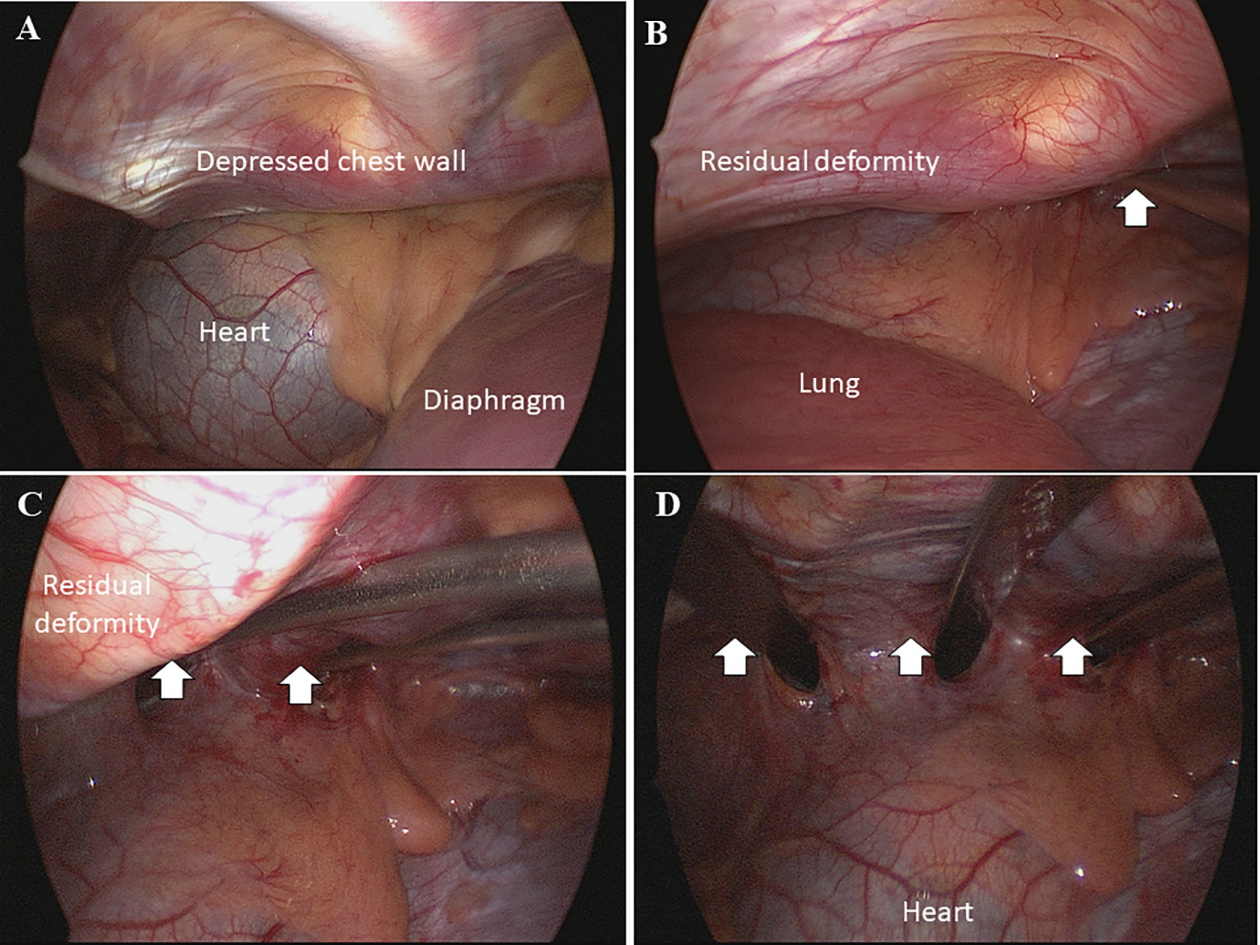
Thoracoscopy-guided repair of a chest wall deformity in a 16-year-old patient. (A) Thoracoscopic view of the deformed chest wall with cardiac compression (B)-(D) Internal changes in the deformed chest wall after the placement of one to three support bars (white arrows).

Most patients were administered thoracic epidural anesthesia intraoperatively and postoperatively for pain control. The patient was placed in a supine position with the arms abducted at about 70° in relation to the body following administration of single-lumen endotracheal tube anesthetic. After confirming the defective area, a small vertical skin incision was made between the anterior and midaxillary lines on each side. After the subcutaneous or submuscular dissections, we entered the pleural cavity at the highest point of the deformity. First, right thoracoscopy, with a 5-mm 15-degree scope inserted into the pleural cavity via the right surgical wound, was performed to directly inspect the mediastinal structures (Fig 2A). Subsequently, a left thoracoscopic inspection was performed via the left surgical wound. Thereafter, a right-to-left mediastinal dissection with the introducer under direct left thoracoscopic visualization was performed, and aberrant vessels found in the mediastinal pleura were avoided to prevent injury during dissection (Fig 2B–2D). To avoid cardiac injury during operation, we routinely used bilateral thoracoscopic inspection when creating the substernal channel. Additionally, if the patient had severe cardiac compression, we used a crane technique to elevate the sunken sternum with a percutaneous steel wire suture passing through the deep point of the sternum. After the substernal tunnel was created, a 28-Fr chest tube was connected to the introducer and retained in the thorax after the introducer was pulled back. A pre-bent Lorenz pectus bar (Zimmer Biomet, Jacksonville, FL, USA) was connected to the chest tube and introduced across the mediastinum. After the pectus bar was rotated and anchored into position, the bar was fixed with either a 1-mm stainless steel wire or with heavy non-absorbing sutures across the adjacent rib and at the ends of the bar and/or the right hinge point. In patients with severe deformities or obvious residual deformities found internally via thoracoscopy, 1 or 2 additional bars were introduced and fixed (Fig 3A–3D). A small-caliber (1/8 inch in diameter) closed-drainage catheter was inserted into each pleural space for drainage of residual air and fluid. After extubation, all patients underwent postoperative radiography and were monitored for 12–24 hours.

### Postoperative care and follow-up

**Fig 4:**
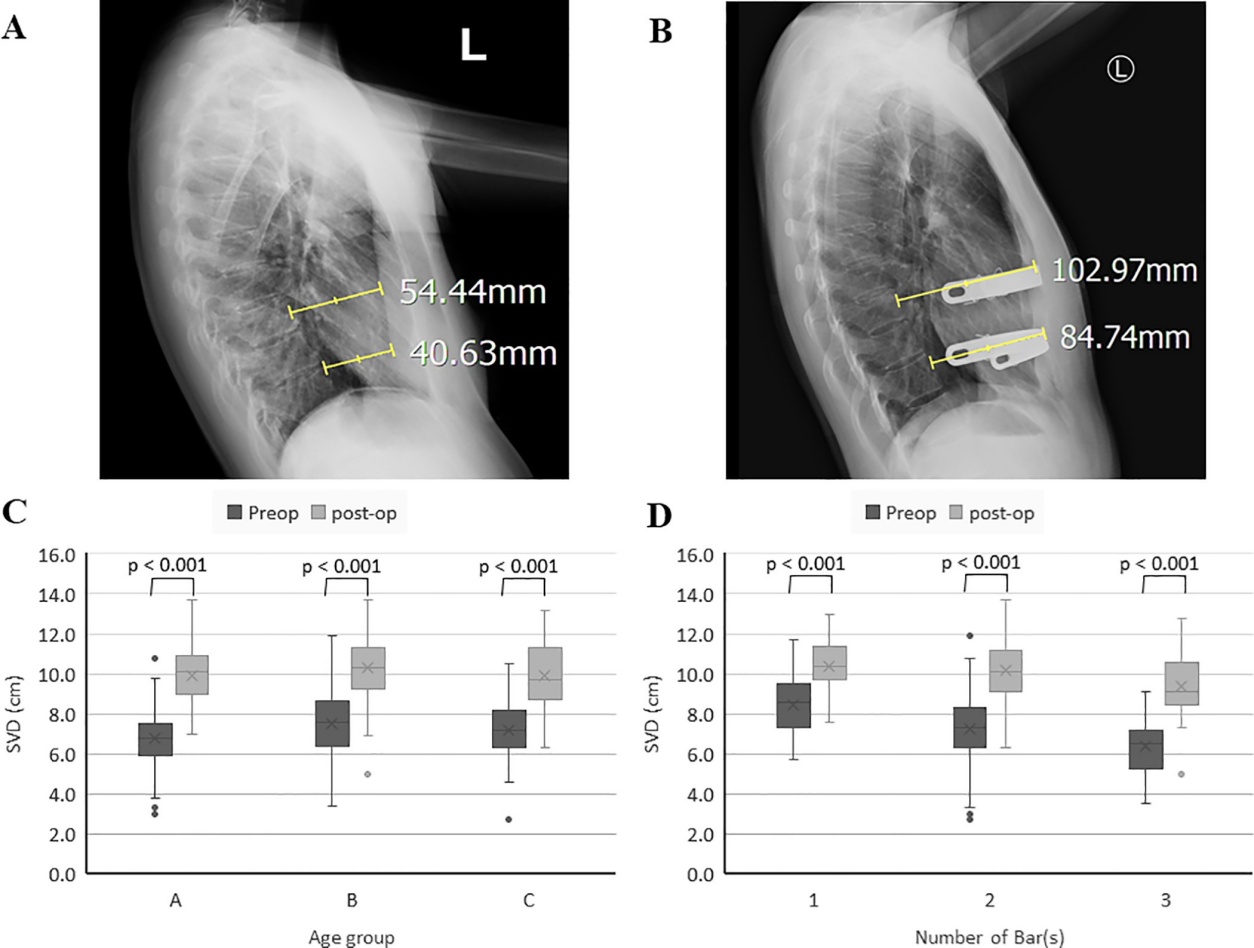
Radiologic changes of the chest wall before and after repair (SVD: Sternovetebral Distance). (A) and (B) Demonstration of the preoperative and postoperative (2 years after surgical repair) SVD in a 23-year-old woman (C) and (D) Box plots of preoperative and postoperative changes in SVD in the different age groups and number of support bar(s).

Generally, postoperative pain was controlled with epidural patient-controlled analgesics (PCA) with opioids (morphine or fentanyl) administrated for 3 days and additional nonsteroidal anti-inflammatory agents. If young patients in whom the epidural catheter was difficult to place or in those who refused to use epidural PCA, opioid PCA for pain relief after surgery was administrated intravenously. Prophylactic antibiotics were administered intravenously, preoperatively and 48 hours postoperatively. Patients were discharged from the hospital when the pain could be managed with oral analgesics. Patients were regularly evaluated via chest radiographs (posteroanterior and lateral views) at 2 weeks, 1 month, 3 months, and 6 months postoperatively, and then once or twice annually thereafter. Postoperatively, cardiopulmonary symptoms were monitored and recorded. SVD values were recorded as an objective index to evaluate the results of the surgical repair (Fig 4A and 4B) [10].

### Statistical analysis

Descriptive data are expressed as mean±SD or range for continuous variables. For continuous data, the Wilcoxon signed-rank test was used to compare medians of variable for before and after comparisons. The Kruskal-Wallis test was used to compare median of variables for between-group comparisons. For categorical variables, the chi-squared or Fisher exact test was used for between-group comparisons. A p-value less than 0.05 was considered statistically significant. Statistical analyses were performed using SPSS software (version 24; IBM, Armonk, NY).

## Results

The demographic and preoperative clinical characteristics of our patients are shown in Table 1. Preoperative symptoms were found to be significantly more common in group C (p<0.001). More than 90% of patients older than 30 years (46/48; 95.8%) had at least one symptom. Progressive chest wall deformity since puberty was present in more than one-third of the patients (113/296; 38.2%). In group C, about two-fifths of the patients had anxiety/depression, which was significantly higher than in other age groups (p = 0.0337). The mean HI and preoperative SVD were 4.0±1.3 and 7.3±1.6 cm, respectively. Valvular regurgitation was significantly more common in group C (p = 0.0454).

**Table 1:** Preoperative demographic and clinical characteristics of 296 patients with pectus excavatum primarily corrected with the modified Nuss procedure.

Characteristic	Group A n = 73	Group B n = 175	Group C n = 48	Test Statistic (df)	p-value
**Mean age, years, mean±SD**	15.6±1.6	23.6±2.6	37.9±6.4	230.12 (2)	<0.001
**Gender, male, n (%)**	65 (89.0)	152 (86.9)	41 (85.4)	0.375 (2)	0.8288
**Symptoms, n (%)**	26 (35.6)	124 (70.9)	46 (95.8)	54.053	<0.001
** Exertional dyspnea**	9 (12.3)	101 (57.7)	38 (79.2)	61.937 (2)	<0.001
** Chest discomfort/pain**	17 (23.3)	117 (66.9)	42 (87.5)	59.253 (2)	<0.001
** Palpitations**	9 (12.3)	46 (28.3)	18 (37.5)	10.482 (2)	0.0052
** Progressive deformity**^**a**^	24 (32.9)	68 (38.9)	21 (45.8)	1.5349 (2)	0.4642
** Scoliosis**	12 (16.4)	37 (21.1)	13 (27.1)	1.9918 (2)	0.3694
** Anxiety/depression**	14 (19.2)	42 (24.0)	19 (39.6)	6.778 (2)	0.0337
** Other disorders**^**b**^	3 (4.2)	9 (5.1)	2 (4.2)	0.134	0.9990
**Cardiac arrhythmia, n (%)**	3 (4.1)	8 (4.6)	3 (6.3)	0.516	0.8590
**Haller index, mean ± SD**	3.9±0.9	4.0±1.4	4.2±1.5	0.89042 (2)	0.6407
**SVD, cm; mean ± SD**	6.8±1.4	7.5±1.6	7.2±1.4	11.937 (2)	0.0525
**Reduced vital capacity, n (%)**	16 (21.9)	50 (28.6)	15 (31.3)	1.5824 (2)	0.4533
**Mitral valve prolapse, n (%)**	13 (17.8)	36 (20.6)	12 (25.0)	0.9158 (2)	0.6326
**Valvular regurgitation, n (%)**	14 (19.2)	52 (29.7)	19 (39.6)	6.0987 (2)	0.0454

^a^ Progression of chest deformity noted after puberty

^b^ Including esophageal vascular ring (1), patent ductus arteriosus (1), spontaneous pneumothorax (10), and bronchiectasis (2)

Perioperative characteristics and overall complications are summarized in Table 2. The operative time was 81.4±14.6 min and was significantly longer in group C (p = 0.0394). The average estimated blood loss was <15 (11.7±5.7) mL in all age groups (p = 0.5654). The number of implanted bars was not significantly different between the age groups (p = 0.8664). All patients were regularly followed up for at least 6 months postoperatively with a follow-up time of 28.6±11 months, and the overall complication rate was 6.8% (20 patients). The complication rate was higher in group C, but non-significantly (p = 0.4164). No mortality or major complications, such as cardiac perforation, massive hemothorax, pericardial effusion, or bar infection, occurred. The most common complication was bar displacement requiring reoperation (3.4%). No patient had pneumothorax. Two patients had pneumonia during hospitalization and were managed with antibiotics. Four patients developed delayed pleural effusion, requiring pleural drainage or short-term steroid administration. Two patients had delayed wound infection occurring at about 3 months postoperatively and were managed with debridement and administration of oral antibiotics. All complications occurred in 3 months postoperatively. More than 6 months postoperatively, two patients still had intractable pain despite being administered analgesics and narcotics. Regarding the clinical characteristics with respect to the different numbers of bars implanted (Table 3), no difference in the average age distribution was found (p = 0.6260). HI and preoperative SVD were significantly severe in patients with multiple bars (p = <0.001 and <0.001, respectively). The mean hospital stay was longer but non-significantly for patients with three bars (p = 0.06). For evaluation of the risk factors of the complication, multivariate logistic regression analysis with odds ratio (OR) and adjusted OR are presented in Table 4. Based on multivariate logistic regression analysis, we found that the complication was associated with Haller index (adjusted OR = 1.2935, p = 0.0317) under controlling confounding factors.

**Table 2:** Perioperative data and overall morbidity of the study groups.

Characteristic	Total 296	Group A n = 73	Group B n = 175	Group C n = 48	Test Statistic (df)	p-value
**Operative time, min, mean±SD**	81.4±14.6	79.6±14.7	80.9±14.3	85.5±14.7	6.4559 (2)	0.0394
**Implanted bar(s), n (%)**					1.314	0.8664
** 1**	28	7 (9.6)	18 (10.3)	3 (6.3)		
** 2**	240	59 (82.8)	142 (81.1)	39 (81.3)		
** 3**	28	7 (9.6)	15 (8.6)	6 (12.5)		
**Estimated blood loss, cc, mean±SD**	11.7±5.7	11.2±5.3	11.7±5.6	12.4±6.6	1.1405 (2)	0.5654
**Complications** ^**a**^**, n (%)**	20 (6.8)	3 (4.1)	12 (6.9)	5 (10.4)	1.856	0.4164
** Cardiac perforation**	0 (0)	0 (0)	0 (0)	0 (0)		
** Reoperation for bleeding**	0 (0)	0 (0)	0 (0)	0 (0)		
** Pneumothorax**	0 (0)	0 (0)	0 (0)	0 (0)		
** Pneumonia**	2 (0.7)	1 (1.4)	0 (0)	1 (2.1)		
** Pericardial effusion**	0 (0)	0 (0)	0 (0)	0 (0)		
** Pleural effusion** ^**b**^	4 (1.4)	0 (0)	3 (1.7)	1 (2.1)		
** Bar infection**	0 (0)	0 (0)	0 (0)	0 (0)		
** Bar displacement** ^**c**^	10 (3.4)	1 (1.4)	6 (3.4)	3 (6.3)		
** Wound infection**	2 (0.7)	0 (0)	2 (1.1)	0 (0)		
** Prolonged pain** ^**d**^	2 (0.7)	1 (1.4)	1 (0.6)	0 (0)		
**Mean hospital stay, days, mean±SD**	6.4±1.7	5.8±1.2	6.3±1.7	6.8±1.7	2.4007 (2)	0.3011
**Postoperative SVD, cm, mean±SD**	10.1±1.4	9.9±1.4	10.3±1.4	9.9±1.6	3.9876 (2)	0.1362

^a^ Overall complications after a mean follow-up of 28.6 (range 6–48) months

^b^ Pleural effusion needing thoracocentesis or pleural drainage

^c^ Bar displacement requiring reoperation

^d^ Administration of analgesics and narcotics after more than 6 months

**Table 3:** Comparison of clinical characteristics based on the numbers of bars inserted.

Characteristic	Total 296	One bar n = 28	Two bars n = 240	Three bars n = 28	Test Statistic (df)	p-value
**Age, years, mean±SD**	23.9±7.7	22.2±6.1	24.0±7.9	24.6±7.6	0.93679 (2)	0.6260
**Haller index, mean±SD**	4.9 ± 1.8	3.7 ± 0.5	4.5 ± 1.5	5.4 ± 3.1	36.329 (2)	<0.001
**Preoperative SVD, cm, mean±SD**	7.3 ± 1.6	8.4 ± 1.3	7.3 ± 1.6	6.4 ± 1.4	23.318 (2)	<0.001
**Operative time, min, mean±SD**	81.4±14.8	59.5±12.3	79.9±6.5	115.0±11.3	113.18 (2)	<0.001
**Estimated blood loss, cc, mean±SD**	11.7±5.7	9.1±4.8	11.3±5.0	18.2±7.6	35.233 (2)	<0.001
**Complications, n (%)**	20 (6.8)	2 (7.1)	16 (6.8)	2 (7.1)	0.275	0.999
** Pneumonia**	2 (0.7)	0 (0)	2 (0.8)	0 (0)		
** Pleural effusion**	4 (1.4)	0 (0)	3 (1.3)	1 (3.6)		
** Bar displacement**	10 (3.4)	2 (7.1)	7 (2.9)	1 (3.6)		
** Wound infection**	2 (0.7)	0 (0)	2 (0.8)	0 (0)		
** Prolonged pain**	2 (0.7)	0 (0)	2 (0.8)	0 (0)		
**Mean hospital stay, days, mean±SD**	6.4±1.7	5.2±1.4	6.2±1.5	7.5±2.6	5.6826 (2)	0.06
**Postoperative SVD, cm, mean±SD**	10.1 ± 1.5	10.4 ± 1.4	10.2 ± 1.5	9.3 ± 1.6	8.5705 (2)	0.0136

**Table 4:** Summary of multivariate logistic regression for risk factors of the complication.

Factor	OR	Adjusted OR	95% CI	p-value
**Age, years**	1.0388	1.0332	0.9773–1.0864	0.2193
**Sex**	0.6742	0.3177	0.0297–1.4839	0.2243
**Operative time, min**	1.0225	1.0139	0.9801–1.0452	0.3951
**Haller index**	1.3108	1.2935	1.0330–1.6981	0.0317

Abbreviation: OR = odds ratio, CI = confidence interval

The postoperative SVD values on the last visit were recorded, and all age groups had significantly improved SVD values compared to the preoperative values (A: 9.9±1.4 vs 6.8±1.5 cm, p<0.001; B: 10.3±1.5 vs 7.5±1.7 cm, p<0.001; C: 9.9±1.6 vs 7.2±1.2 cm, p<0.001) (Fig 4C). In all groups, the postoperative SVD values on the last visit were significantly improved compared to the preoperative SVD values (one bar: 10.4±1.4 cm vs 8.4±1.3 cm, p<0.001; two bars: 10.2±1.5 cm vs 7.3±1.6 cm, p<0.001; C: 9.3±1.6 cm vs 6.4±1.4 cm, p<0.001) (Fig 4D). In general, the SVD after postoperative follow-up with mean of 28.6 months showed significant improved compared with that before surgery (7.3±1.6 and 10.1± 2.8 cm, respectively; p<0.001).

## Discussion

In recent decades, PE has been considered not only as a cosmetic problem but also as a psychosocial and/or physiological concern, especially in elderly patients [5,16–18]. The common cardiopulmonary symptoms are chiefly caused by long-term compression of cardiopulmonary structures and progressive impairment of chest wall flexibility. During puberty, overgrowth of the costal cartilages might occur, but the mechanism underlying this phenomenon is still unclear. In our data, about one-third of patients had a clinical history of progressive chest wall deformity since their adolescence. Recent studies recommend that the procedure be performed in patients aged between 12 and 16 years, when the chest wall is relatively mature, although not fully ossified [19,20]. In our study, disabling cardiopulmonary symptoms were significantly more frequent in older patients. Valvular regurgitation was also observed in older patients, with a significant trend, and might be associated with cardiopulmonary symptoms. Most of the research results indicate that mitral valve prolapse is related to pectus excavatum [16,17,19], but whether valve regurgitation is related to pectus excavatum is not clear. Our results found that the patients with pectus excavatum might be associated with valve regurgitation, but their correlation needs further elucidate. Surgery could also be considered in young patients for cosmetic reasons or to prevent the sequelae of cardiopulmonary compression later in life. Otherwise, only older patients would undergo surgical repair because of cardiopulmonary problems.

Surgical repair is the standard treatment for PE, and a minimally invasive technique was introduced by Dr. Nuss in 1998 [3]. The acceptability and popularity of this procedure increased rapidly as it required less radical resection and had good cosmetic and functional results [3, 21–23]. Relative contraindications for the use of the Nuss bar include the presence of significant primary cardiac dysfunction (not related to the pectus deformity), mental or neurological conditions resulting in patients being unwilling or incapable of following instructions, metal sensitivity reactions and concurrent complex congenital abnormalities [24]. Severe intrathoracic adhesion due to previous infection or intrathoracic surgery could also be carefully evaluated.

Initially, the use of one bar was reported for most patients with PE. As the procedure became more popular, the use of more than one bar became common, especially in adult patients [4–6]. However, the number of bars used depended primarily on the severity of the gross chest wall deformity and the surgeons’ experience. We performed a modified Nuss repair technique using a bilateral thoracoscopy-assisted approach in more than 600 patients between July 2005 and July 2013, and initially, two stainless-steel bars were placed in about 25% of the patients to correct the deformity above the heart area [9,10,11]. Since the technique was effective and safe, we started performing advanced repair with modified techniques in patients with profound and wide deformity of the chest wall, using multiple bars under thoracoscopic guidance (S1 Video). The criteria for the advanced repair were assessed through internal inspection of the changes of the deformed chest wall using a thoracoscope and by referring to the gross external appearance during the repair. In our study, more than 90% of the patients had two or three bars placed to correct the deformed chest wall, and there was no significant difference between the adolescent adult patients. HI and SVD were significantly severe in patients with more than one bar, indicating that the placement of more bars might be needed in patients with severe PE. The surgical time to place three bars was significantly longer. Although the estimated blood loss was significantly greater for the placement of three bars, it was non-notable. Postoperatively, SVD values significantly improved after at least 6 months in all groups. However, owing to the small number of patients with one bar (28 patients) and three bars (28 patients), postoperative complications were not evaluated. In the multivariate logistic regression analysis for complications, we found that the complication of the procedure was associated with the severity of pectus deformity, but not with age or number of bars.

Postoperative complications of the Nuss procedure have been reported in several studies [4–9,12,25]; common complications included postoperative pneumothorax, pleural effusion, bar displacement requiring reoperation (1.7%-6.6%), pericardial effusion, hemothorax requiring blood transfusion or reoperation, cardiac injury, and infectious complications, including bar infection, pneumonia, or wound infection. The complication rate was mostly reported to be higher in adults [4, 7–9]. Modifications for improving the efficacy and safety of the Nuss procedure include the use of split-lung ventilation with CO_2_ insufflation [3,5], forced sternal elevation [5,26], bilateral application of the thoracoscope bilaterally [9,27], improvement in fixation of bars [3,5,26], routine pleural drainage [9,28], and multiple bar support [4–6]. Cardiac injury is the most serious complication. To avoid cardiac injury during operation, some techniques to force sternal elevation (such as crane technique) were illustrated [5,25]. Since we used bilateral thoracoscopic surgery to increase the safety of the procedure, the crane technique was used in only 12 procedures. In our current modifications, we still perform bilateral thoracoscopy with the thoracoscope inserted into the incision made for the bar, without performing other incisions for the thoracoscope. The hypoinflation of the lungs during mediastinal dissection allowed excellent visualization of each pleural cavity. Carbon dioxide insufflation was not used in our procedures. The aberrant vessels in the mediastinal pleura were not injured, resulting in minimal blood loss. A small-caliber catheter was inserted into the pleural cavities to allow closed drainage of pleural air and fluid for 2 days; hence, no postoperative pneumothorax occurred in any patient. Routine chest tube drainage was not necessary. Infectious complications associated with the implant are a rare but serious problem. The Nuss procedure is a clean surgery, but if the intrathoracic implant is contaminated, infection can be difficult to control. In our study, only two patients had delayed wound healing because of infection after almost 3 months postoperatively. Prophylactic antibiotic treatment was administered preoperatively and 48 hours postoperatively.

Many subjective questionnaires or objective examinations (CT, cardiopulmonary exercise test, echocardiography, PFT, etc.) are used to evaluate the postoperative outcomes depending on the purpose of the follow-up. We evaluated the postoperative outcomes of the surgical correction by measuring the SVD values on the lateral chest radiographs, as this is relatively objective and can be easily performed and repeated [10,29]. Our results demonstrated that the postoperative SVD values of the patients significantly improved after the repair. Therefore, the Nuss procedure could improve a deformed chest wall, and SVD measurement could be a useful postoperative assessment. Implant removal was recommended for all patients who were followed up more than 3 years postoperatively. The long-term outcome of our modified approach requires further evaluation in the future.

The study had some limitations. It was a retrospective, one-center and one-surgeon experience. Though our patients were followed up with a mean of 28.6 months, the long-term outcomes after removal of bar(s) should be investigated. Furthermore, although significant anatomic improvement, demonstrated by postoperative change on SVD and subjective improvement of symptoms by medical records, was obtained, further objective tests are needed to elucidate the functional outcomes after surgical repair.

## Conclusion

The modified Nuss repair with bilateral thoracoscopic guidance for adolescent and adult patients with PE resulted in fewer cardiopulmonary complications. The aggressive correction of the deformed chest wall using multiple bars was also performed easily and safely. This approach could be an alternative method for the selection of placed bars in adolescent and adult patients.

## Supporting information

S1 Checklist STROBE statement—checklist of items that should be included in reports of observational studies. (DOCX)

S1 Video Video demonstration for modified nuss procedure. 1. A 16 years old man with severe pectus excavatum underwent the modified Nuss procedure. 2. Single skin incision was made bilaterally. 3. The right mini-thoracoscopic inspection via the right surgical wound was done at first. 4. A right-to-left mediastinal dissection was completed under the left thoracoscopic visualization. 5. A pre-bending bar was passed through the mediastinum and rotated. 6. Using 2nd and 3rd metal bars for aggressive repair the residual deformity of the chest wall was done upon the internal thoracoscopic guidance and on gross appearance. 7. After fixation of bars, each small-caliber close drainage catheter was inserted into the pleural spaces before wounds closure.
(MP4)
